# A case of successful removal of 15 magnetic beads causing gastric penetration by endoscopic full-thickness resection

**DOI:** 10.1055/a-2621-3491

**Published:** 2025-07-10

**Authors:** Huihui Zhou, Baohua Yu, Yaowen Zhang

**Affiliations:** 1 562122 Department of Gastroenterology, Affiliated Hospital of Jining Medical University, Jining, China; 2 562122 Department of Pediatric Surgery, Affiliated Hospital of Jining Medical University, Jining, China; 3 562122 Endoscopy Department, Affiliated Hospital of Jining Medical University, Jining, China

The incidental finding on the chest X-ray ( Fig. 1 **a** ) in a 2-year-old boy was as follows: a string of beads-like radiopaque shadow in the left upper abdomen. Abdominal computed tomography ( Fig. 1 **b** ) was as follows: abnormal density with artifacts in the right upper abdomen. The patient may have ingested the magnetic beads 6 days earlier without discomfort. Esophagogastroduodenoscopy showed ( Fig. 2 **a** ) a semicircular chain of magnetic beads in the stomach, with both ends penetrating the gastric wall ( Video 1 ). Initially, eight magnetic beads were successfully removed using rat-tooth forceps, and concurrent X-ray imaging ( Fig. 2 **b, c** ) showed seven remaining magnetic beads, six of which were located outside the gastric wall. Despite repeated attempts to remove the remaining beads, they were deeply embedded and surrounded by tissue, with both ends already perforating the gastric wall, preventing successful removal. One exposed magnetic bead was targeted for endoscopic full-thickness resection. A dual knife was used to incise the mucosal surface, followed by progressive dissection with the IT2-Knife to cut through the muscularis propria until the magnetic bead was fully exposed and reformed into a ring. The bead was then removed with a snare. In total, 15 magnetic beads were removed. A follow-up X-ray showed no residual foreign bodies. After achieving sufficient hemostasis, to avoid delayed perforation, nine metal clips and one nylon loop were used to close the wound ( Fig. 2 **d–h** ). The patient recovered well and experienced no discomfort after eating.

**Fig. 1:**
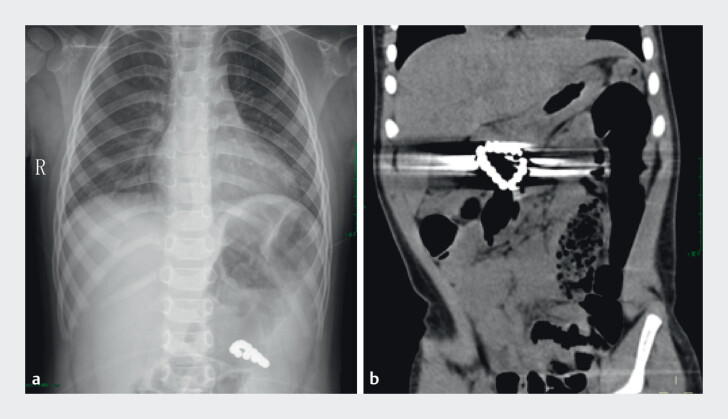
**a** The X-ray shows a ring-shaped radiopaque foreign body in the upper left abdomen. **b** The computed tomography suggests a suspicious ring-shaped foreign body within the intestine, with no evidence of active gastrointestinal perforation.

**Fig. 2:**
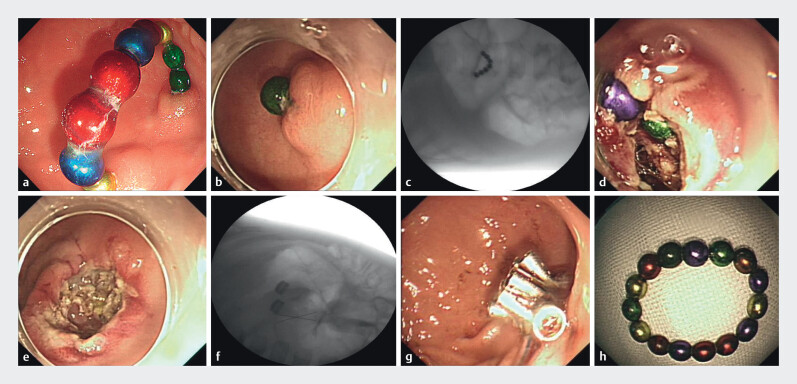
**a** Endoscopy revealed a semicircular chain of magnetic beads, with both ends having penetrated the gastric wall. **b** Endoscopic image after the removal of eight magnetic beads from the stomach. **c** X-ray showing remaining magnetic beads. **d** Endoscopic full-thickness resection was performed. **e** Endoscopic full-thickness resection site after the foreign body removal. **f** X-ray shows no residual foreign body. **g** Postoperative wound closure. **h** The removed magnetic beads.

A case of successful removal of 15 magnetic beads causing gastric penetration by endoscopic full-thickness resection.Video 1

X-rays showing that the magnet was above the pelvic level should be considered to be within the accessible range of the upper gastrointestinal tract for endoscopy 1 2 . Conventional wisdom suggests that deeply embedded magnets are more difficult to remove and often require surgical intervention in the event of fistulae and perforation 3 . Ultimately, we were able to successfully remove the foreign body through endoscopy, thus avoiding more invasive surgical intervention.

Endoscopy_UCTN_Code_TTT_1AO_2AL

## Notes

Endoscopy E-Videos https://eref.thieme.de/e-videos
*E-Videos* is an open access online section of the journal *Endoscopy* , reporting on interesting cases and new techniques in gastroenterological endoscopy. All papers include a high-quality video and are published with a Creative Commons CC-BY license. Endoscopy E-Videos qualify for HINARI discounts and waivers and eligibility is automatically checked during the submission process. We grant 100% waivers to articles whose corresponding authors are based in Group A countries and 50% waivers to those who are based in Group B countries as classified by Research4Life (see: https://www.research4life.org/access/eligibility/ ). This section has its own submission website at https://mc.manuscriptcentral.com/e-videos .
